# Supplementary material for: Establishing a pure antiferroelectric PbZrO3 phase through tensile epitaxial strain
Source: Nat Commun. 2025 Jul 16;16:6536. doi: 10.1038/s41467-025-61867-y (PMC12264186; doi:10.1038/s41467-025-61867-y)
Supplement: Supplementary file 1 — Supplementary Information [file 41467_2025_61867_MOESM1_ESM.pdf]

Table of contents

Supplementary Fig. 1 2
Supplementary Fig. 2 3
Supplementary Fig. 3 4
Supplementary Fig. 4 5
Supplementary Fig. 5 6
Supplementary Fig. 6 7
Supplementary Fig. 7 8
Supplementary Fig. 8 9
Supplementary Fig. 9 10
Supplementary Fig. 10 11
Supplementary Fig. 11 12
Supplementary Note 1 13
Supplementary Fig. 12 14

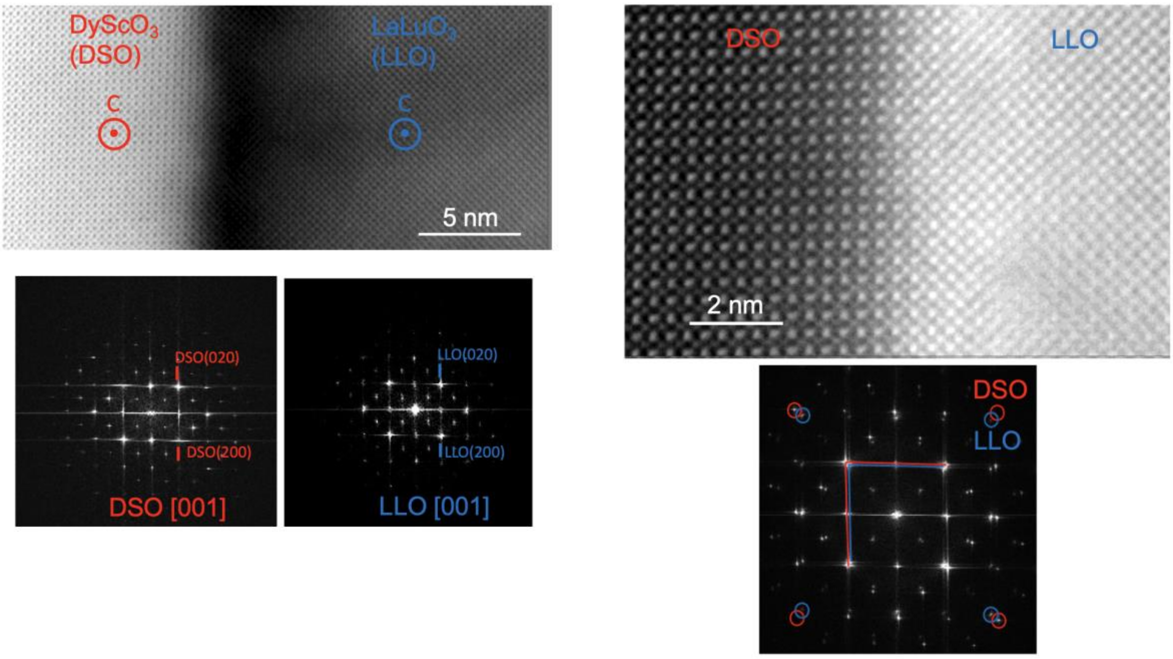

Supplementary Figure 1. Structural characterizations of $LaLuO_3$ thick films grown on $DyScO_3$(110). (Left) BF-STEM image of $LaLuO_3$ on $DyScO_3$ with the corresponding FFTs of the two materials underneath. (Right) HAADF-STEM image showing a high crystalline quality of the interface and the FFT emphasizes the epitaxial relationship between the two orthorhombic perovskites with a monoclinic distortion associated to the (110) growth. The [001] orthorhombic axis is the zone axis for both $DyScO_3$ and $LaLuO_3$.

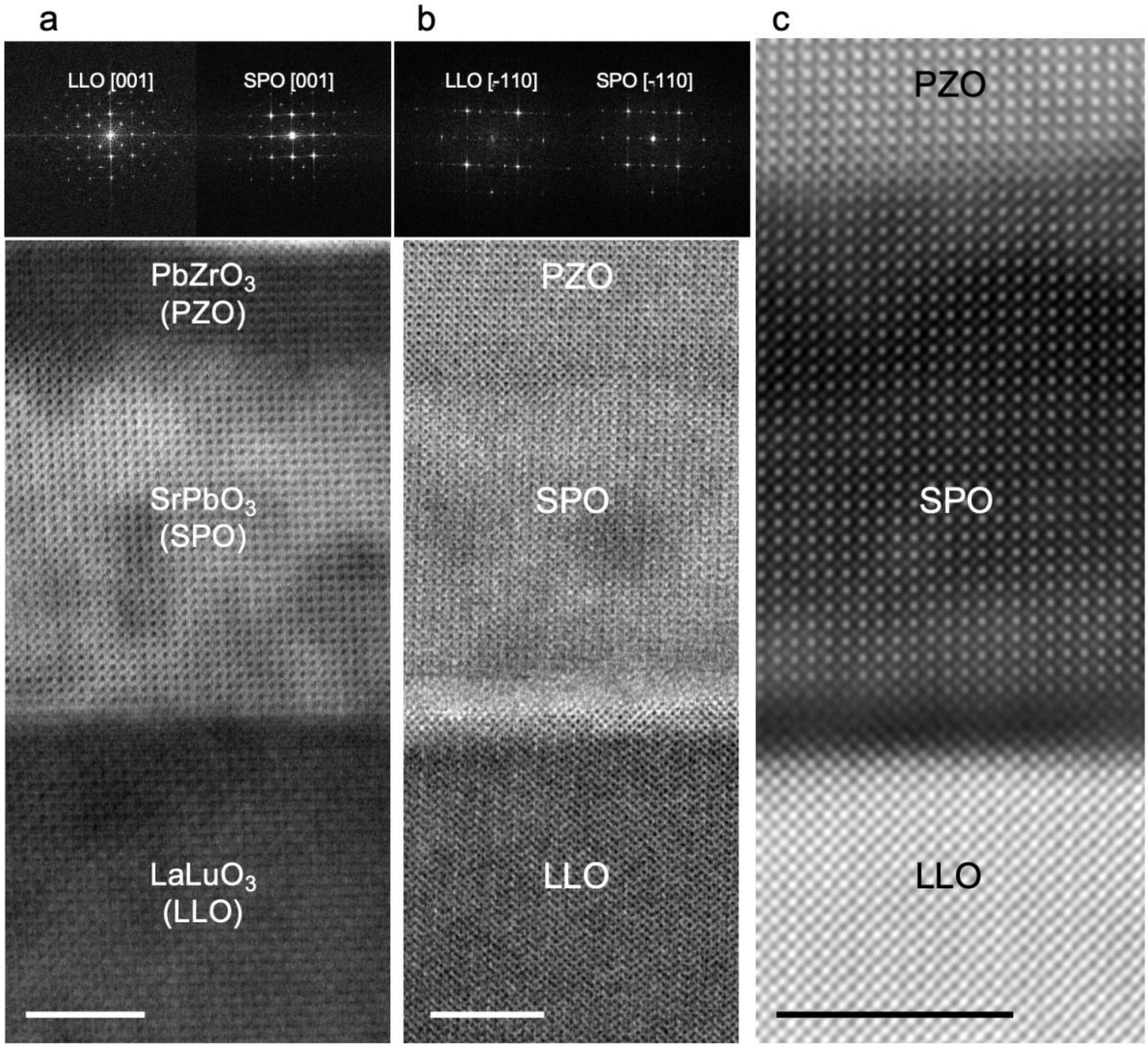

Supplementary Figure 2. STEM investigations of the $SrPbO_3$ layer. a, ABF-STEM image on a cross-section area with the zone axis parallel to the c_o axis of $DyScO_3$. FFTs from the $LaLuO_3$ and $SrPbO_3$ layers indicate that they both have an orthorhombic structure with their c_o axes parallel to that of the $DyScO_3$ substrate. b, BF-STEM image on a cross-section area with the zone axis perpendicular to the c_o axis of $DyScO_3$. FFTs for $LaLuO_3$ and $SrPbO_3$ indicate that their c_o axes lie in the film plane. c, HAADF-STEM image on a cross-section area showing the $LaLuO_3/SrPbO_3/PbZrO_3$ interfaces. Despite some partial amorphization at the $LaLuO_3/SrPbO_3$ interface, the continuity between the planes is clearly seen between the three layers with a sharp Z-contrast inversion between the $SrPbO_3$ and $PbZrO_3$, as the heavy Pb element jumps between the B-site and the A-site. Horizontal scale bars are 5 nm.

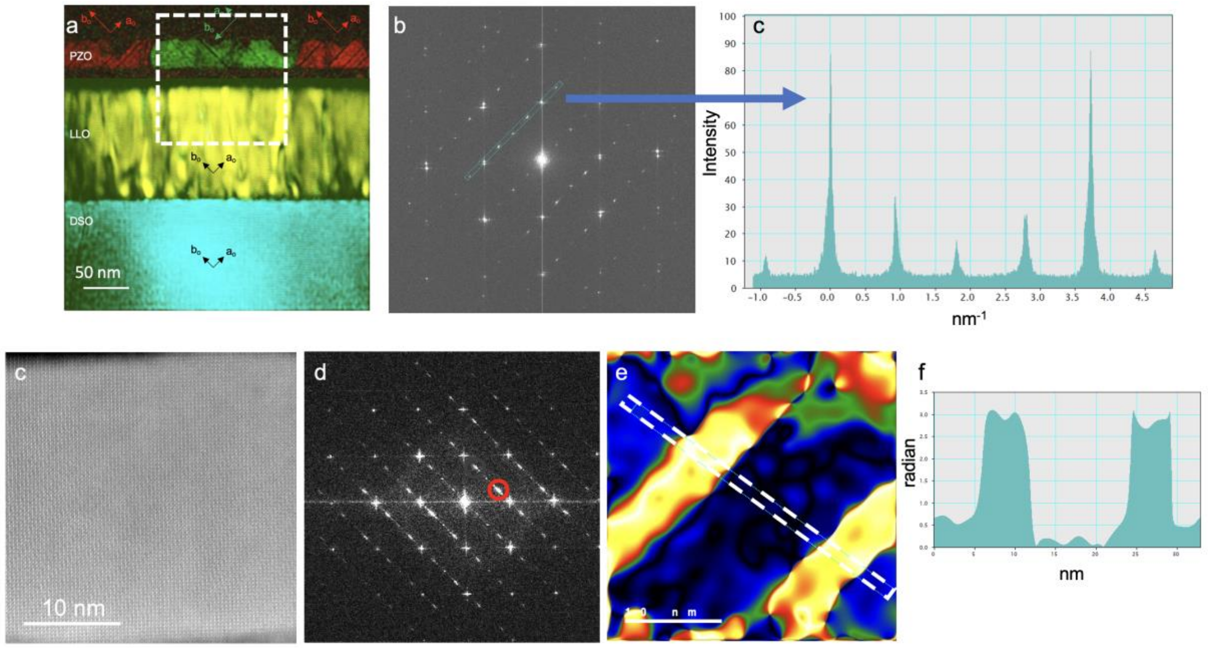

Supplementary Figure 3. a, Same image as in the main text Figure 1b. b, FFT from the squared area in (a), located in the green $PbZrO_3$ domain. c, Intensity profile from the FFT pattern, showing a well-defined ¼ super periodicity corresponding to a pure P*bam* phase. c, HAADF-STEM image of an area containing several “stripe lines” in a $PbZrO_3$ layer of 30 nm. d, Corresponding FFT pattern; the red circle indicates the ¼ periodicity selected for phase analysis. e, Phase distribution of the super periodicity; the blue and yellow regions are separated by a π-phase shift. f, Phase profile across the antiphase domain and translation boundaries (in radians), taken from the boxed area in (c).

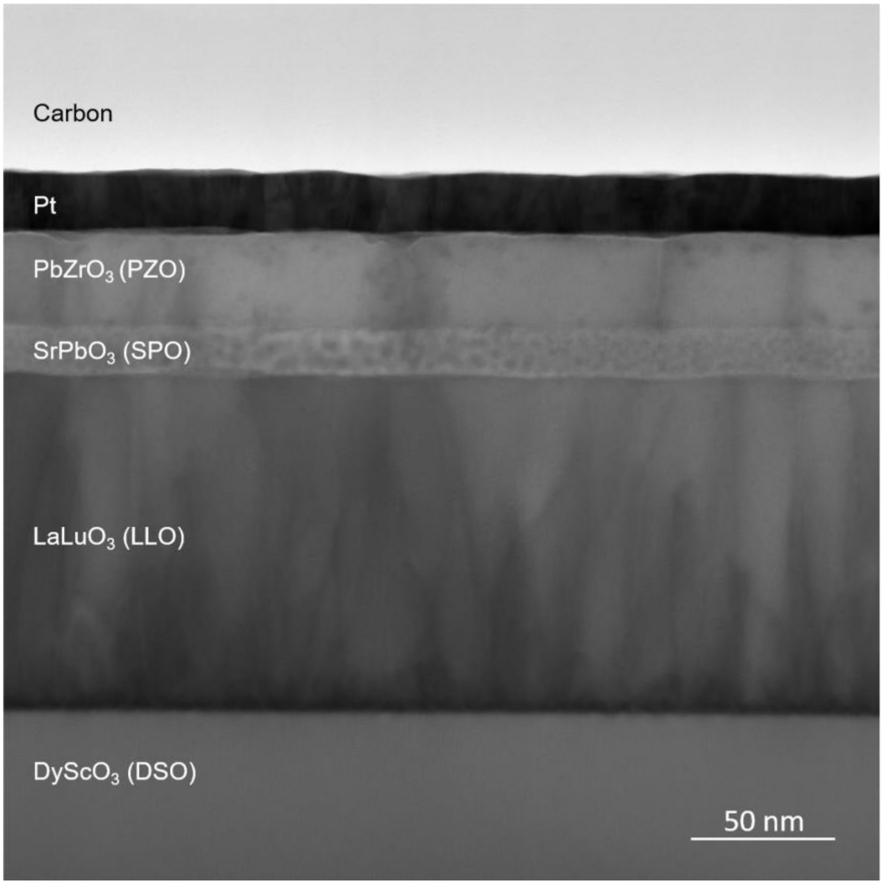

Supplementary Figure 4. Bright field STEM image on the cross-section specimen of the $PbZrO_3$ (30 nm) / $SrPbO_3$ (19 nm) / $LaLuO_3$ (108 nm) epitaxial stack grown on $DyScO_3(110)_o$. The corresponding geometrical phase analysis is displayed in Figure 1c-d of the manuscript.

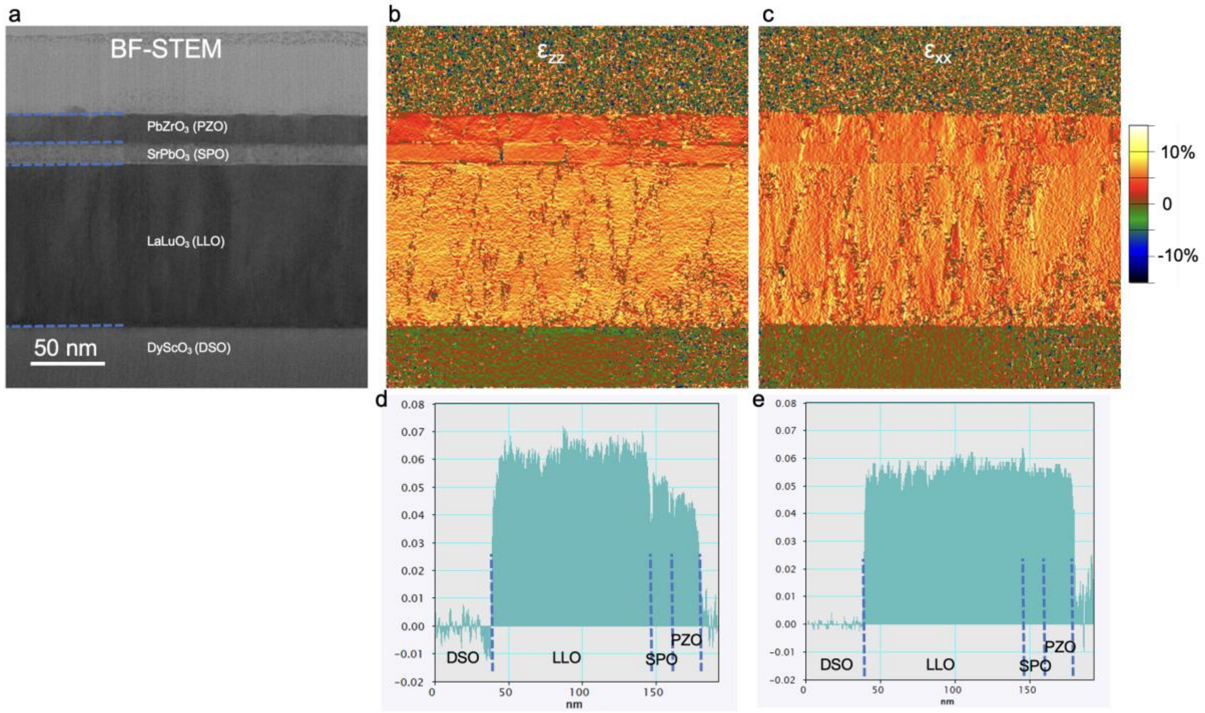

Supplementary Figure 5. a, Bright field STEM image (left) on the cross-section specimen of the $PbZrO_3$ (20 nm) / $SrPbO_3$ (15 nm) / $LaLuO_3$ (110 nm) epitaxial stack grown on $DyScO_3(110)_o$. Geometrical phase analysis gives rise to the b, out-of-plane (middle) and c, in-plane (right) deformations at the unit-cell level. The corresponding out-of-plane and in-plane deformation profiles are displayed in (d) and (e), respectively. The constant in-plane deformation between the three layers suggests that $PbZrO_3$ is coherently strained by the $LaLuO_3$ film.

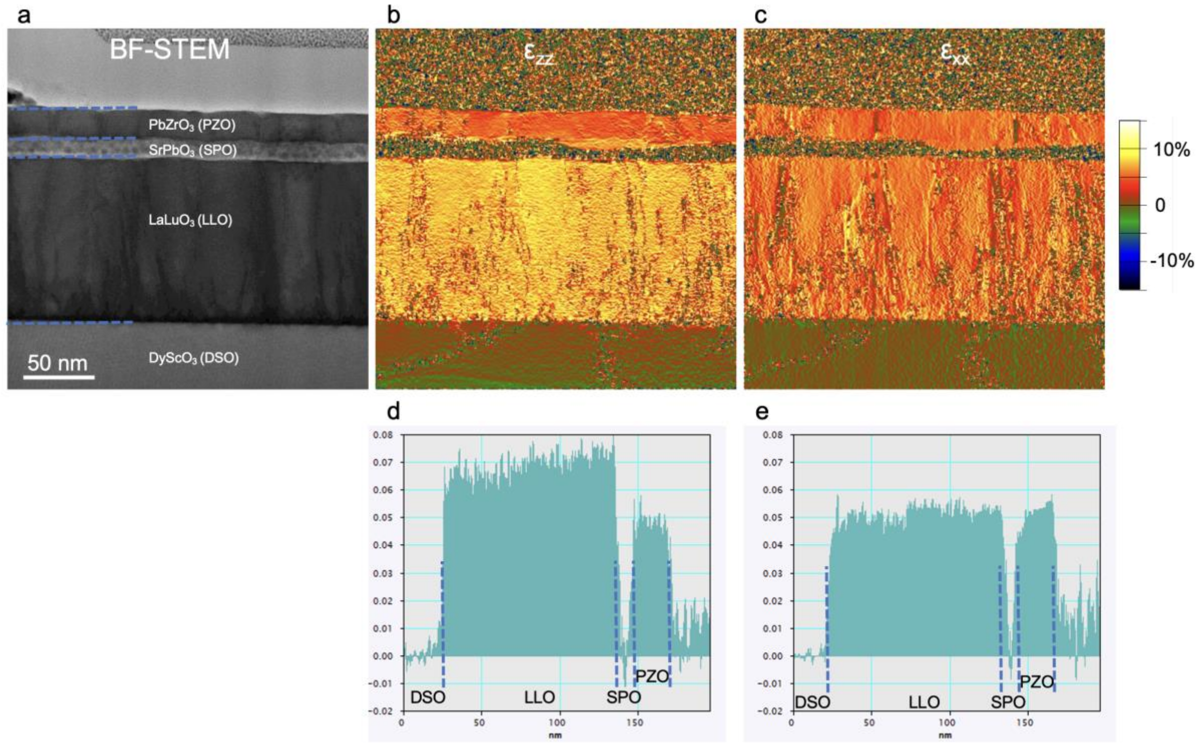

Supplementary Figure 6. a, Bright field STEM image (left) on another cross-section specimen of the $PbZrO_3$ (20 nm) / $SrPbO_3$ (15 nm) / $LaLuO_3$ (110 nm) epitaxial stack grown on $DyScO_3(110)_o$. In this area, the $SrPbO_3$ electrode has been strongly amorphized by the electron beam. Geometrical phase analysis gives rise to the b, out-of-plane (middle) and c, in-plane (right) deformations at the unit-cell level. The corresponding out-of-plane and in-plane deformation profiles are displayed in (d) and (e), respectively. The constant in-plane deformation between the layers suggests that $PbZrO_3$ is still coherently strained by the $LaLuO_3$ film.

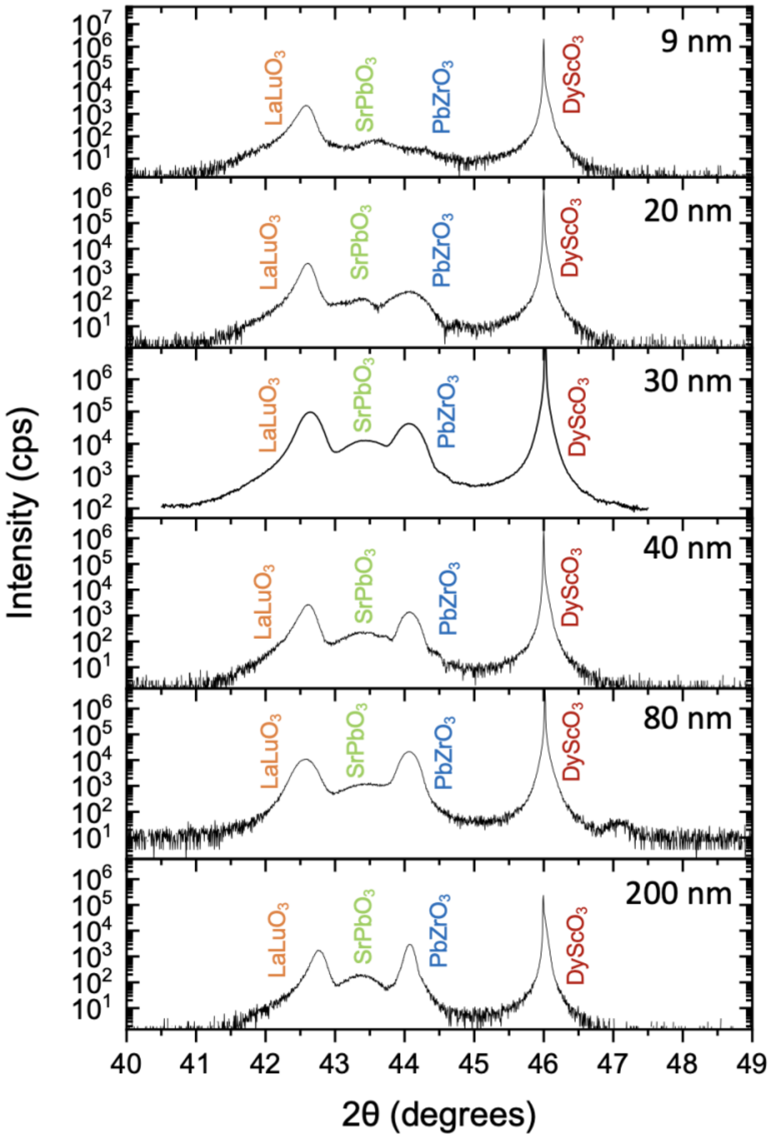

Supplementary Figure 7. 2θ-ω X-ray diffraction patterns of the $PbZrO_3/SrPbO_3/LaLuO_3$ samples grown on $DyScO_3$, with $PbZrO_3$ film thicknesses varying from 200 nm (bottom) to 9 nm (top).

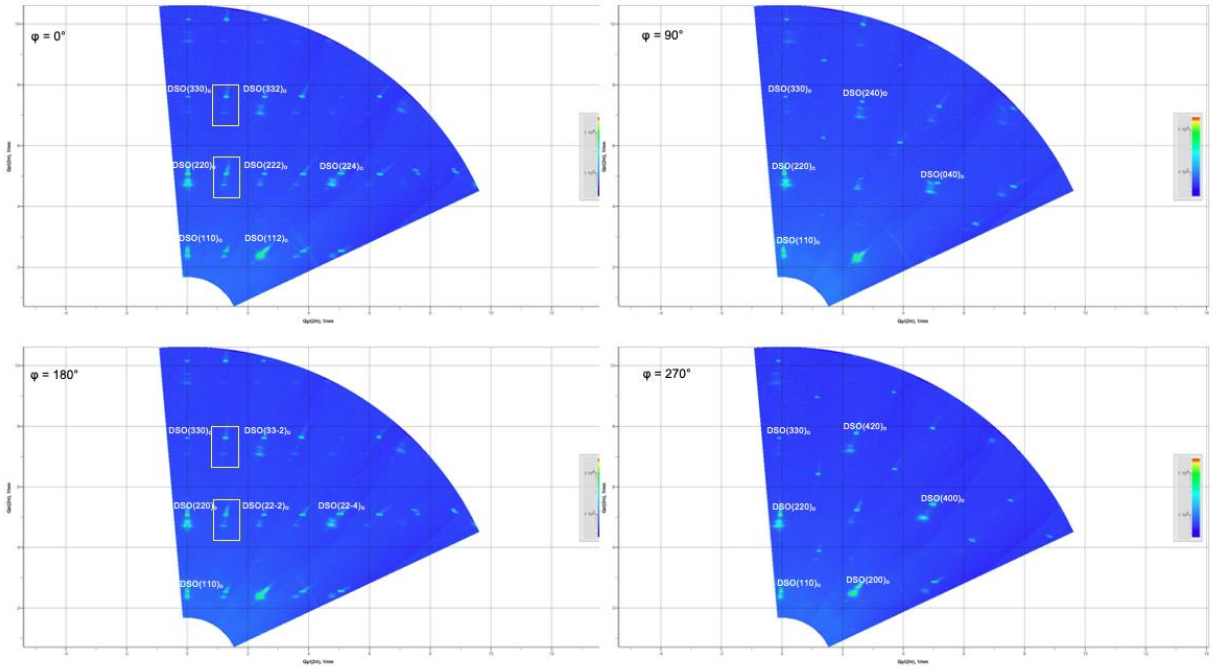

Supplementary Figure 8. Two-dimensional reciprocal space maps of a 40-nm thick $PbZrO_3$ film grown on $SrPbO_3$/$LaLuO_3$ buffered $DyScO_3(110)_o$. The three orthorhombic layers share the same diffraction pattern as $DyScO_3$ along the four scattering planes corresponding to azimuthal angles φ equal to 0°, 90°, 180° and 270°. More precisely, ½{110} peaks are observed at φ equal to 90° and 270°, corresponding to c_o being orthogonal to such scattering planes. Instead ½{100} peaks are observed for φ equal to 0° and 180°, corresponding to c_o lying in such scattering planes (see the yellow rectangles).

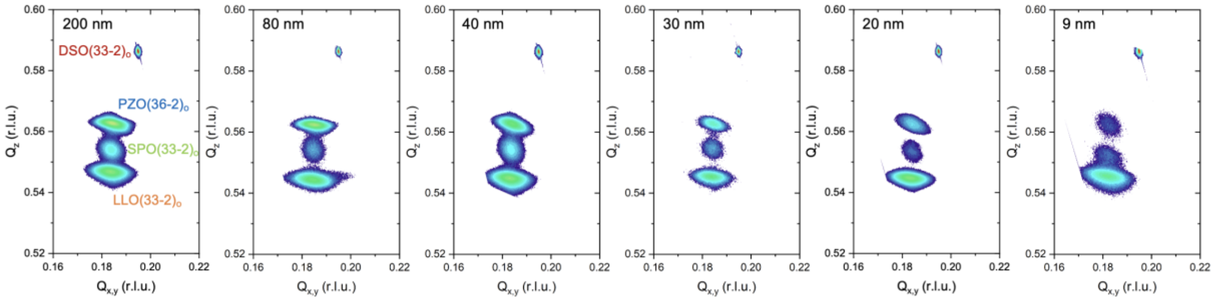

Supplementary Figure 9. Reciprocal space maps around the $(33\bar{2})_o$ $DyScO_3$ peaks. The $PbZrO_3$ film thicknesses are 200 nm, 80 nm, 40 nm, 30 nm, 20 nm, and 9 nm. The three orthorhombic layers of $PbZrO_3$, $SrPbO_3$, and $LaLuO_3$ share the same in-plane $Q_{x,y}$ for all the film thicknesses. In addition, there is no detectable change in the Q_z value of the $PbZrO_3$ peak as a function of thickness, suggesting no strain relaxation.

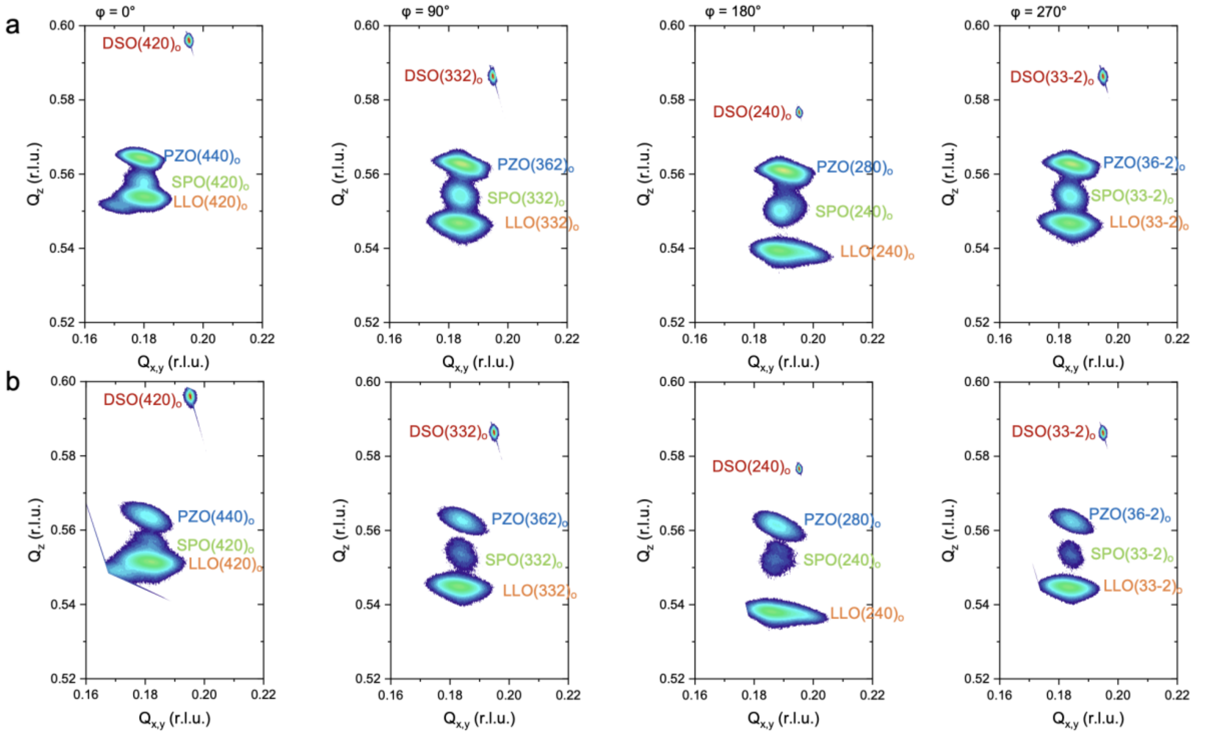

Supplementary Figure 10. Reciprocal space maps taken at φ equal to 0°, 90°, 180°, and 270°, corresponding here to the $(420)_o$, $(332)_o$, $(240)_o$, and $(33\bar{2})_o$ $DyScO_3$ peaks, respectively. The $PbZrO_3$ film thicknesses are 200 nm (a) and 20 nm (b). The three orthorhombic layers of $PbZrO_3$, $SrPbO_3$, and $LaLuO_3$ show symmetric peak positions in Q_z and $Q_{x,y}$ for φ equal to 90° and 270°, as for the $DyScO_3$ substrate peaks. Instead, a shift in Qz is observed between φ equal to 0° and 180° for $PbZrO_3$, $SrPbO_3$, $LaLuO_3$, and $DyScO_3$. This confirms the $(110)_o$ orientation of $SrPbO_3$ and $LaLuO_3$ and $(120)_o$ for $PbZrO_3$ (as the b_o parameter is doubled for the latter), and the c_o axes of the three layers and the $DyScO_3$ are lying in the sample plane and parallel to each other. The two in-plane and the out-of-plane pseudo-cubic cell parameters of $PbZrO_3$ were estimated in Figure 2c from these four maps using: (in green) ∥ $c_{DSO} = \lambda \times (Q_x(362) + Q_x(36\bar{2}))^{-1}$; (in red) ⊥ $c_{DSO} = \lambda \times (Q_x(440) + Q_x(280))^{-1}$; (in dark blue) out-of-plane $= \lambda \times 6 \times (Q_z(440) + Q_z(362) + Q_z(280) + Q_z(36\bar{2}))^{-1}$. λ is the Cu K_α wavelength of the X-rays.

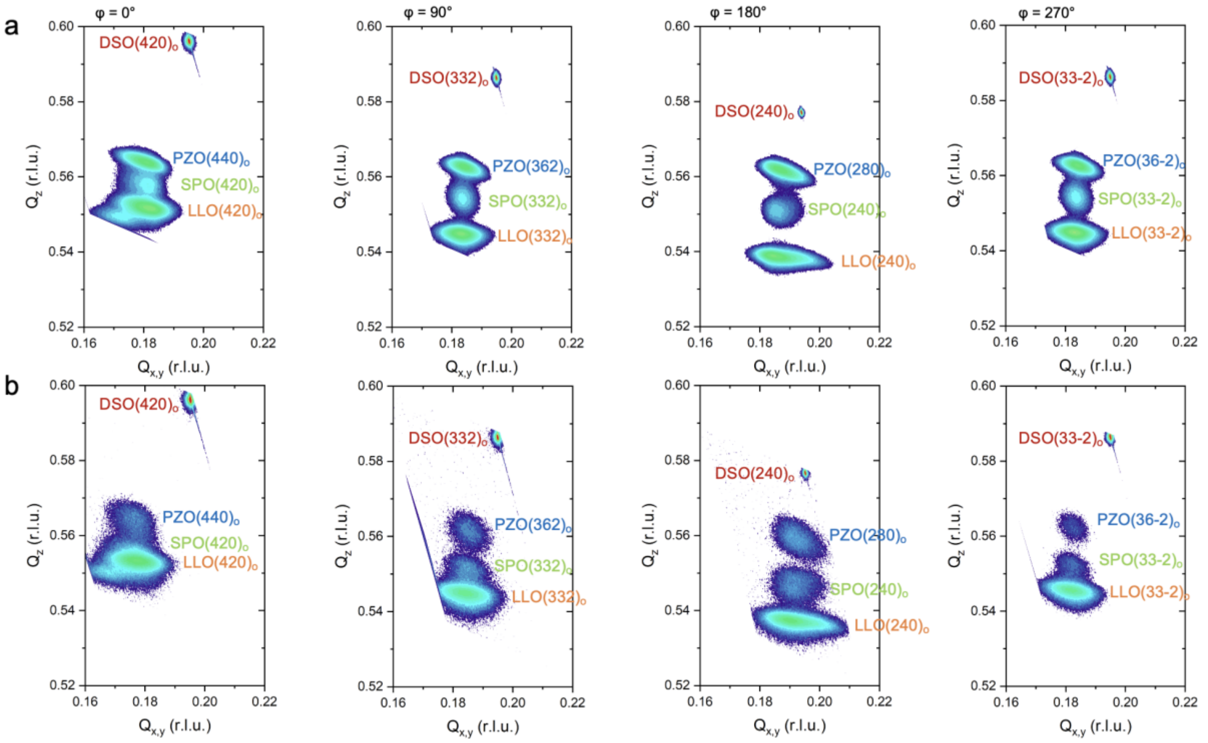

Supplementary Figure 11. Reciprocal space maps taken at φ equal to 0°, 90°, 180°, and 270°, corresponding to the $(420)_o$, $(332)_o$, $(240)_o$, and $(33\text{-}2)_o$ $DyScO_3$ peaks, respectively. The $PbZrO_3$ film thicknesses are 40 nm (a) and 9 nm (b). Same comments as for Supplementary Figure 10.

Supplementary Note 1 – Calculations of the epitaxial strain in $PbZrO_3$

Considering $(120)_o$ $PbZrO_3$ on top of $(110)_o$ $SrPbO_3/LaLuO_3$ with all the three orthorhombic layers having their c_o parallel, we can estimate the epitaxial strain imposed by the orthorhombic layers on $PbZrO_3$. Taking the bulk cell parameters of $PbZrO_3$, $LaLuO_3$, and $SrPbO_3$, the strain is estimated along c_o or perpendicularly to c_o as: $\frac{x_{LLO,SPO}-x_{PZO}}{x_{PZO}}$ %.

	$PbZrO_3$	$LaLuO_3$	$SrPbO_3$
ao (Å)	5.882	5.810	5.852
bo (Å)	11.783	6.013	5.969
co (Å)	8.228	8.373	8.324
// c_o (Å)	4.114	4.187	4.162
⊥ c_o (Å)	4.163	4.181	4.180
strain $PbZrO_3$ // c_o (%)		1.76	1.17
strain $PbZrO_3$ ⊥ c_o (%)		0.44	0.41

Supplementary Table 1. Bulk orthorhombic unit-cells of $PbZrO_3$, $SrPbO_3$, and $LaLuO_3$ and estimated strain on $PbZrO_3$.

Hence, we clearly see from Supplementary Table 1 that the in-plane strain imposed by $LaLuO_3$ or $SrPbO_3$ on $PbZrO_3$ is anisotropic, with +0.4% and +1.2-1.8%, perpendicularly or along c_o, respectively. This is due to the fact that the lattice mismatch between $PbZrO_3$ and $SrPbO_3$ or $LaLuO_3$ is larger along c_o, than along a_o or b_o.

To calculate the epitaxial strain experimentally measured in the $PbZrO_3$ thin films with various thicknesses, we compared the experimental cell parameters estimated from the X-ray diffraction reciprocal space maps with the bulk parameters of $PbZrO_3$ (Supplementary Table 2). The evolution of these parameters with the film thickness is displayed in Figure 2c. In the last three rows of Supplementary Table 2, we report the epitaxial strain along the three axes, calculated as: $\frac{x_{film}-x_{bulk}}{x_{bulk}}$ %.

film thickness	9	20	30	40	80	200	bulk
⊥ c_o (Å)	4.177	4.178	4.178	4.177	4.178	4.171	4.163
// c_o (Å)	4.187	4.186	4.184	4.184	4.184	4.179	4.114
out-of-plane (Å)	4.113	4.108	4.106	4.106	4.109	4.107	4.163
strain ⊥ c_o (%)	0.35	0.38	0.37	0.34	0.37	0.20	
strain // c_o (%)	1.76	1.74	1.71	1.71	1.70	1.59	
strain out-of-plane (%)	-1.18	-1.31	-1.37	-1.36	-1.30	-1.34	

Supplementary Table 2. Experimental pseudo-cubic cell parameters estimated from X-ray diffraction reciprocal space maps and calculated epitaxial strain considering $(120)_o$ orientation.

The experimental strain values reported in this Supplementary Table 2 demonstrate that the films are under anisotropic tensile strain in the plane, resulting in an out-of-plane compression of the unit-cell. From the comparison with Supplementary Table 1, it appears that the $LaLuO_3$ buffer layer is imposing this amount of anisotropic tensile strain to the $PbZrO_3$. The thickness dependence of the experimental epitaxial strain of the $PbZrO_3$ films is displayed in Figure 2d.

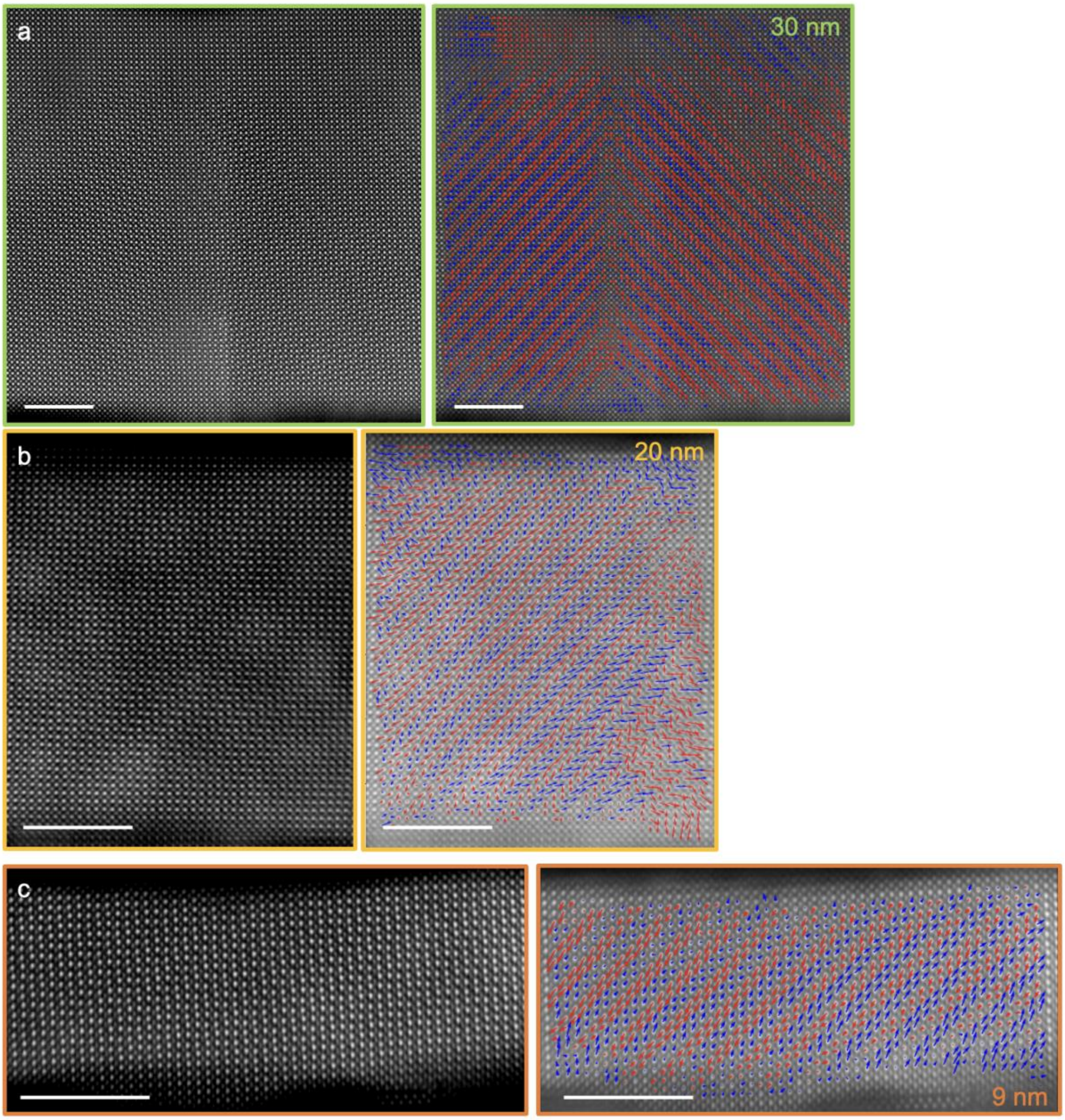

Supplementary Figure 12. High resolution HAADF-STEM images for a, the 30-nm-thick $PbZrO_3$ film, b, the 20-nm-thick $PbZrO_3$ film and c, the 9-nm-thick $PbZrO_3$. In all the images, the zone axis is parallel to the c_o axis of $DyScO_3$. The resulting dipoles are represented as coloured arrows in the right images (same images as in Figure 4). Horizontal scale bars are 5 nm.
